# Arthroscopic partial repair for massive rotator cuff tears: does it work? A systematic review

**DOI:** 10.1186/s40798-019-0186-z

**Published:** 2019-04-11

**Authors:** Michael-Alexander Malahias, Lazaros Kostretzis, Efstathios Chronopoulos, Emmanouil Brilakis, Grigorios Avramidis, Emmanouil Antonogiannakis

**Affiliations:** 1 3rd Orthopaedic Department, Hygeia Hospital, Erythrou Stavrou 4, 151 23 Marousi, Athens Greece; 2 HKF Zentrum, ATOS Klinik, Heidelberg, Germany; 3 2nd Orthopaedic Department, School of Medicine, National and Kapodistrian University of Athens, Agias Olgas 3, 14233 Nea Ionia, Athens Greece

**Keywords:** Shoulder arthroscopy, Arthroscopic partial repair, Medialized repair, Massive rotator cuff tear, Systematic review

## Abstract

**Background:**

While arthroscopic complete repair of massive rotator cuff tears (MRCT) back to their anatomic footprint is preferential, there are cases where this type of repair is not applicable due to the contraction of the torn tendons. In such cases, a non-anatomic incomplete or partial repair can be performed. A number of clinical studies have investigated the clinical and functional outcomes of arthroscopic partial repair for irreparable MRCT. To our knowledge, no systematic review has been published yet to synthetically evaluate these results.

**Methods:**

Two reviewers independently conducted the search in a PRISMA-compliant systematic way using the MEDLINE/PubMed database and the Cochrane Database of Systematic Reviews. These databases were queried with the terms “arthroscopy”[MeSH Terms] OR arthroscopic surgical procedure [Text Word (tw)] AND massive rotator cuff tears [tw] AND arthroscopic partial repair [tw].

**Results:**

From the 55 initial studies, we finally chose 11 clinical studies which were eligible to our inclusion-exclusion criteria. The mean modified methodology Coleman score was 58/100, whereas it ranged from 41/100 to 78/100. In total, 643 patients were included in this review. All postoperative mean clinical and functional subjective scores, as well as muscle strength of patients treated with arthroscopic partial repair, were found significantly improved, when compared with the respective mean preoperative values. The rate of structural failure of the partial repair, as it was estimated by postoperative imaging modalities, was 48.9%. The overall reoperations’ rate was 2.9% regarding the patients who were treated with partial repair.

**Conclusions:**

Arthroscopic partial repair might be a safe and effective alternative treatment for irreparable contracted MRCT, where a complete repair cannot be performed. The methodological quality of the relevant, available literature is low to moderate; therefore, further studies of higher quality are required to confirm these results.

## Key Points

- Arthroscopic partial repair might be a safe and effective salvage solution in cases where an arthroscopic complete repair of massive rotator cuff tears cannot be performed.
- The methodological quality of the relevant, available literature is low to moderate; therefore, further studies of higher quality are required to confirm our results.

## Background

The understanding of rotator cuff pathology and healing continues to evolve, beginning with emerging descriptions of the anatomic footprint and natural history of rotator cuff tears [1]. Shoulder anatomy, pathology, and biomechanics place unique stress on the rotator cuff tendons during sports activity [2], whereas smoking, hypercholesterolemia, and genetics have all been shown to influence the development of rotator cuff tearing [3].

Depending on the degree of cuff pathology, acromioplasty, debridement of partial cuff tears, and repair of full-thickness tears are usually successful in those who fail a rehabilitation program [4]. Complete repair, if possible, is the optimal treatment for full-thickness rotator cuff tears [5]. On the other hand, the management of massive, irreparable rotator cuff tears (MRCT) is challenging and associated with high failure rates, since there are no current consensus or definitive guidelines concerning the optimal surgical treatment for this devastating condition [6]. Arthroscopic options include rotator cuff repair, partial cuff repairs, tendon allografts or xenografts, decompression, débridement, biceps tenotomy, tenodesis, and tendon transfers [7]. The treatment modality specifically chosen for the massive, irreparable rotator cuff tear must be tailored to the individual patient, their needs and expectations, and their ability to comply with intensive rehabilitation [8].

While arthroscopic complete repair of MRCT back to their anatomic footprint is preferential, there are cases where this type of repair is not applicable due to the contraction of the torn tendons. In such cases, a non-anatomic incomplete or partial repair can be performed. Arthroscopic partial repair is indicated for young patients when the muscle is still trophic with a fatty infiltration less than 3, according to the MRI-based Fuchs classification [9]. It can be combined with a tendon transfer like latissimus dorsi (or alternatively lower trapezius) in irreparable posterosuperior tears or pectoralis major (or alternatively latissimus dorsi) in anterosuperior cuff tears [10].

A number of clinical studies have investigated the clinical and functional outcomes of arthroscopic partial repair for irreparable MRCT [11–21]. To our knowledge, no systematic review has been published yet to synthetically evaluate these results.

Our aims were twofold: (1) to summarize failure rates and clinical/functional/radiographic outcomes associated with the arthroscopic partial repair as the method of treatment for symptomatic irreparable MRCT and (2) to characterize the methodological quality of the relevant, available literature. Our hypothesis was that arthroscopic partial repair would be proven a safe and effective treatment for this type of lesions where no complete repair can be done.

## Materials and methods

Two reviewers (MAM, LK) independently conducted the search in a systematic way according to the Preferred Reporting Items for Systematic Reviews and Meta-Analyses (PRISMA) using the MEDLINE/PubMed database and the Cochrane Database of Systematic Reviews. These databases were searched using the terms “arthroscopy”[MeSH Terms] OR arthroscopic surgical procedure [Text Word (tw)] AND massive rotator cuff tears [tw] AND arthroscopic partial repair [tw]. To maximize the search, backward chaining of reference lists from retrieved papers was also undertaken. A preliminary assessment of only the titles and abstracts of the search results was initially performed. The second stage involved a careful review of the full-text publications.

Inclusion criteria were clinical studies investigating adult patients, diagnosed with symptomatic MRCT, who were treated with arthroscopic partial repair, and who had a minimum of 12 months clinical follow-up (with clinical tests and/or scores). These studies should have been written in English or German as full-text articles and they should have been published by March 1, 2018 (end of our search).

We excluded from our review all studies which were not dealing with partial repair (other means of operative or nonoperative treatment), non-arthroscopic procedures (open or mini-open), studies about reparable massive or non-massive RCT, trials with follow-up less than 12 months, studies without clinical/functional outcome variables, abstracts, editorial comments, case reports, corrigenda, technical notes, literature reviews, preclinical studies, and papers not written in English or German.

Differences between reviewers were discussed until agreement was achieved. In cases of disagreement, the senior author (EA) had the final decision. The two reviewers independently extracted data from each study and assessed variable reporting of outcome data. Descriptive statistics were calculated for each study and parameters analyzed. The “quality assessment” of the studies for methodological deficiencies, as a common alternative to “risk of bias” [22], was examined by the modified Coleman methodology score [23]. The methodological quality of each study and the different types of detected bias were assessed independently by each reviewer and then they were combined synthetically. Selective reporting bias like publication bias was not included in the assessment. The primary outcome measure was the failure rate leading to reoperation and the clinical, functional, and radiographic outcomes. The secondary outcome was the quality assessment of the studies with the use of the modified Coleman methodology score.

## Results

**Fig. 1:**
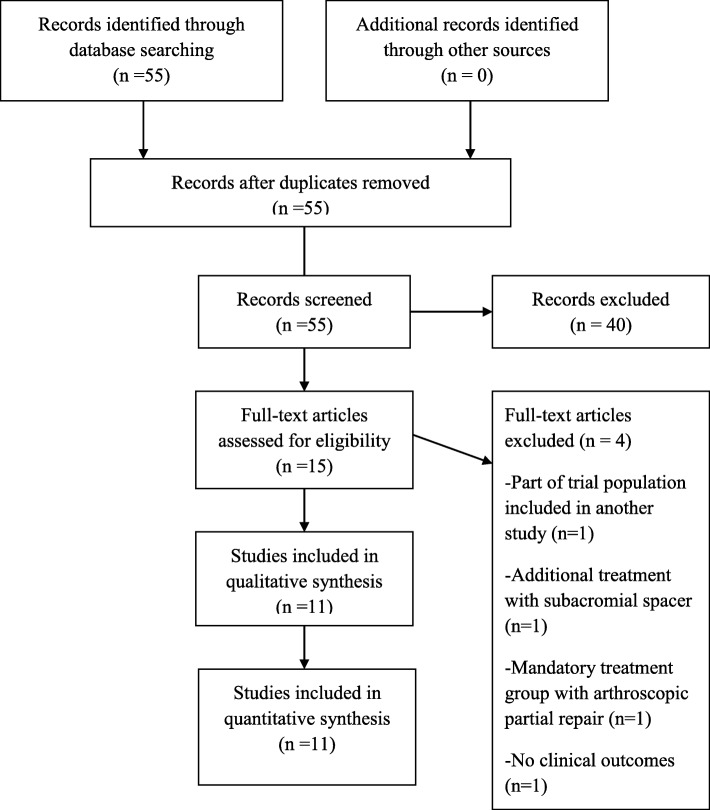
Flow chart of study selection according to PRISMA guidelines for reporting systematic reviews and meta-analyses

From the 55 initial studies, we finally chose and assessed 11 clinical studies which were eligible to our inclusion-exclusion criteria [11–21]. We excluded all the irrelevant studies (16), technical notes (6), trials not referring to massive rotator cuff tears (5), reviews of the literature (4) (not relevant to our review), papers concerning only full repair (4), mini-open procedures (2), studies dealing with massive rotator cuff tears treated with subacromial biodegradable spacers (2) or greater tuberoplasty (1), trials regarding pigmented villonodular synovitis (1), case reports (1), papers without any clinical outcome (1), and papers written in Chinese (1). A summary flowchart of our literature search according to PRISMA guidelines can be found in Fig. 1.

### Level of evidence and study’s design

All studies included in this review were published between 2010 and 2016 [11–21] (Table 1).

**Table 1:** Year of study, design, and type of surgery for studies included in the review

Study	Year	Prospective/retrospective design	Type of surgery
Berth et al. [11]	2010	Prospective	Arthroscopic partial repair vs arthroscopic debridement
Cuff et al. [12]	2016	Retrospective	Arthroscopic biceps tenotomy and partial repair
Francheschi et al. [13]	2012	Prospective	Arthroscopic debridement vs arthroscopic partial repair
Godenèche et al. [14]	2016	Prospective	Arthroscopic complete repair vs arthroscopic partial repair
Heuberer et al. [15]	2015	Prospective	Arthroscopic debridement vs arthroscopic partial repair vs arthroscopic complete repair
Holtby et al. [16]	2014	Retrospective	Arthroscopic complete repair vs arthroscopic partial repair
Iagulli et al. [17]	2012	Retrospective	Arthroscopic complete repair vs arthroscopic partial repair
Kim et al. [18]	2012	Retrospective	Arthroscopic partial repair
Mori et al. [19]	2013	Retrospective	Arthroscopic patch graft procedure vs arthroscopic partial repair
Paribelli et al. [20]	2015	Prospective	Arthroscopic-assisted latissimus dorsi tendon transfer vs arthroscopic partial repair
Shon et al. [21]	2015	Retrospective	Arthroscopic partial repair

Six out of the 11 studies of this review (54.5%) had a level of evidence III [13–17, 19], while three studies (27.3%) had a level of evidence IV [12, 18, 21], one study (9.1%) a level of evidence I [11], and another one (9.1%) a level of evidence II [20] (Table 2).

**Table 2:** Level of evidence, modified Coleman methodology score, and high risk of possible bias per study

Study	Study level	Modified Coleman score (0–100)	Risk of bias
Berth et al. [11]	I	78	Selection, performance, detection
Cuff et al. [12]	IV	56	Performance, detection, attrition
Francheschi et al. [13]	III	70	Selection, performance, detection
Godenèche et al. [14]	III	63	Selection, performance, attrition
Heuberer et al. [15]	III	59	Selection, performance
Holtby et al. [16]	III	41	Selection, performance, detection
Iagulli et al. [17]	III	51	Selection, performance, attrition
Kim et al. [18]	IV	53	Performance, attrition
Mori et al. [19]	III	48	Selection, performance, detection, attrition
Paribelli et al. [20]	II	60	Selection, performance, detection
Shon et al. [21]	IV	59	Selection, performance

We found eight comparative studies (72.7%) [11, 13–17, 19, 20] and three non-comparative studies (27.3%) [12, 18, 21], whereas only one trial (9.1%) [11] was randomized and ten trials (90.9%) were non-randomized [12–20]. The most common comparisons were between arthroscopic complete repair and partial repair [14–17] as well as arthroscopic partial repair and debridement [11, 13, 15]. One study compared the arthroscopic partial repair technique with latissimus dorsi transfer [20] and another one with a patch autograft procedure [19] (Table 1).

### Quality of the studies and possible risk of bias

The mean modified methodology Coleman score for methodological deficiencies of the studies was 58/100, whereas it ranged from 41/100 [16] to 78/100 [11] (Table 2).

All studies of this review (100%) were characterized by a possible high risk of performance bias [11–21], while nine studies (81.8%) were found with possible selection bias [11, 13–17, 19–21], six studies (54.5%) with possible detection bias [11–13, 16, 19, 20], and five studies (45.5%) with possible attrition bias [12, 14, 17–19] (Table 2).

### Demographics

Totally, 643 patients were included in this review. From them, 348 patients were treated with an arthroscopic partial repair procedure, while 295 patients underwent another type of treatment (either as control or as study groups). The majority of the patients who were treated with the arthroscopic partial repair technique were males (63.7%). The mean age of the patients who underwent arthroscopic partial repair ranged from 59.4 years [14] to 68 years [15], while the mean age of the patients who were treated with other operative techniques ranged from 62.5 years [20] to 66.5 years [15]. The mean follow-up of the patients who underwent arthroscopic partial repair ranged between 16 [11] and 93.6 months [13], whereas the mean follow-up of the patients who were treated with other operative techniques ranged from 24 months [11, 16, 17] to 93.6 months [13] (Table 3).

**Table 3:** Number of shoulders per study, sex, mean age, mean follow-up, clinical/functional, and imaging outcome variables used per study

Study	Number of shoulders	Sex (males/females)	Mean age (years)	Mean follow-up (months)	Clinical outcome scales	Imaging outcome evaluation
Berth et al. [11]	42 (partial: A, 21; debridement: B, 21)	A, 15/6 B, 16/5	A, 62.5 ± 2.3 B, 64.3 ± 3.4	A, 16 ± 3 B, 24 ± 2	Constant score, DASH score, ROM	Ultrasonography
Cuff et al. [12]	28	19/9	65.2 (60–90)	71.1 (60–90)	ASES score, SST score, VAS score, ROM	Radiographic (Hamada stage)
Francheschi et al. [13]	68 (debridement: A, 34; partial: B, 34)	A, 22/12 B, 25/9	62 (47–76)	93.6 ± 27.6	ROM, modified UCLA, RC-QOL	Not evaluated
Godenèche et al. [14]	73 (complete: A, 50; partial: B, 23)	52/21	59.4 ± 8.8	41 (29–55)	Constant score, subjective shoulder value, strength	Ultrasonography
Heuberer et al. [15]	68 (debridement: A, 23; partial: B, 22, complete: C:23)	40/28 (A, 11/12; B, 14/8; C, 15/8)	66.5 ± 7.2 (A, 66.5; B, 68; C, 65)	45	Constant score, ROM, VAS, subjective shoulder value, qDASH, satisfaction rate	MRI, ultrasonography
Holtby et al. [16]	122 (partial: A, 73; complete: B, 49)	A, 48/25 B, 33/16	A, 67 ± 9 B, 64 ± 9	24	ASES score, relative Constant-Murley score, short western Ontario rotator cuff index, ROM, strength	Not evaluated
Iagulli et al. [17]	97 (86 evaluated) (partial: A, 45; complete: B, 52)	Not reported	A, 64.5 ± 9.5 B, 63.4 ± 12.2	24 (10–40)	UCLA score	Not evaluated
Kim et al. [18]	27	Not reported	62.3 (54–72)	41.3 (36–52)	UCLA score, Constant score, ROM, strength	Radiographic (acromiohumeral distance)
Mori et al. [19]	48 (patch: A, 24; partial: B, 24)	A, 17/7 B, 10/14	A, 65.9 ± 8.9 B, 65.4 ± 9.2	A, 35.5 ± 8.6 B, 35.7 ± 7.0	Constant score, ASES score, UCLA score, VAS score, ROM, strength	MRI
Paribelli et al. [20]	40 (tendon transfer: A, 20; partial repair: B, 20)	A, 13/7 B, 11/9	A, 62.5 (45–77) B, 64.9 (47–78)	33.6 (12–60)	Modified UCLA score, ROM, RC-QOL,VAS score, strength	Not evaluated
Shon et al. [21]	31	17/14	65.9 ± 6.5	40 ± 14.9	VAS-pain score, ASES score, simple shoulder test score, patients’ satisfaction	Radiographic (Hamada classification)

### Reoperation rate

The overall reoperation rate was 2.9% for patients who were treated with partial repair. The most common reason for reoperation was severe glenohumeral osteoarthritis (1.1%) and the second most common reason was failure of the repair and persistent pain (0.9%). The most common type of reoperation was reverse shoulder arthroplasty (1.1%) (Table 4).

**Table 4:** Preoperative and postoperative clinical and functional mean scores per study, complications, and take-home message

Study	Preoperative scores	Postoperative scores	Complications	Take-home message
Berth et al. [11]	A: Constant 36.9 DASH 64.6 ROM Abd 97.7° Add 28° IR 67.5° ER 41.7°	A: Constant 58.2 DASH 23.8 ROM Abd 144° Add 37.2° IR 79.5° ER 47°	A: 1 patient with persistent pain was reoperated arthroscopic debridement)	In cases of massive rotator cuff rupture, early and mid-term results of partial repair were slightly superior to those of arthroscopic debridement alone.
	B: Constant 29.9 DASH 69.5 ROM Abd 93.5° Add 28° IR 49.5° ER 40.5°	B: Constant 40.7 DASH 35.3 ROM Abd 103.5° Add 35.2° IR 71.6° ER 42.7°	B: 1 patient developed severe glenohumeral arthritis and was treated with shoulder hemiarthroplasty	
Cuff et al. [12]	ASES 46.6 SST 5.6 VAS 6.9 ROM FF 168° ER 38° IR 84% full IR	ASES 79.3 SST 9.1 VAS 1.9 ROM FF 154° ER 39° IR 80% full IR	3 patients elected for revision to reverse shoulder arthroplasty	The treatment of massive rotator cuff tears with partial arthroscopic rotator cuff repair and biceps tenotomy results in midterm subjective satisfaction in the majority of patients.
Francheschi et al. [13]	A: Mod UCLA 7.6 VAS 6.7 RC-QOL N.A. ROM ER 42.9° IR 37.8° FF 104.1°	A: Mod UCLA 21.4 VAS 1.5 RC-QOL 61.8 ROM ER 42.9° IR 37.8° FF 104.1°	Not reported	In the surgical treatment of irreparable rotator cuff tears, arthroscopic debridement associated \with acromioplasty and bursectomy and partial repair of rotator cuff tear are both effective in reducing symptoms, with the latter also offering higher functional outcomes.
	B: Mod UCLA 8.6 VAS 6.8 RC-QOL N.A. ROM ER 40.6° IR 40° FF 111.5°	B: Mod UCLA 28.8 VAS 1.8 RC-QOL 71.2 ROM ER 50.5° IR 68.7° FF 163.5°		
Godenèche et al. [14]	A: Constant 30.8 Strength 1.1 kg SSV N.A.	A: Constant 79.7 Strength 5.3 kg SSV 79.2	Not reported	The repair of massive rotator cuff tears with partial or complete repair results in equivalent Constant scores improvement.
	B: Constant 32.2 Strength 1.5 kg SSV N.A.	B: Constant 75.3 Strength 3.6 kg SSV 70.2		
Heuberer et al. [15]	N/A	A: Constant: 65.8 Q-DASH: 24.1	A: 1 patient with revision to reverse shoulder arthroplasty due to pain	Arthroscopic debridement, partial rotator cuff repair, and complete rotator cuff repair are effective in treating massive rotator cuff tears. Complete rotator cuff repair shows better short-term results.
		B: Constant: 67.5 Q-DASH: 20.5	B: 2 patients with postoperative infections: treated with arthroscopic debridement and lavage, 1 patient with anchor loosening: arthroscopic anchor removal	
		C: Constant: 80.3 Q-DASH: 7.0 Patients satisfied: A: 87%, B: 86%, C: 91%	C: 1 patient with re-tear and revision to reverse shoulder arthroplasty and 1 patient with infection	
Holtby et al. [16]	A: ASES 42.6 CMS 44.0 ShortWORC 34.6 ROM Flex 110.1° Abd 102.7° ER 36.2° Strength 3.8	A: ASES 71.4 CMS 73.7 ShortWORC 62.7 ROM Flex 129.5° Abd 121.3° ER 42.8° Strength 5.9	Not reported	The partial repair of massive rotator cuff tears showed a statistically significant improvement in ROM, strength and disability scores. However, the results were slightly inferior compared to complete repair.
	B: ASES 51.0 CMS 47.6 ShortWORC 38.9 ROM Flex 119.9° Abd 107.2° ER 44.4° Strength 4.8	B: ASES 82.8 CMS 87.9 ShortWORC 79.4 ROM Flex 153.4° Abd 142.5° ER 49.1° Strength 9.9		
Iagulli et al. [17]	A: UCLA 12.1	A: UCLA 29.5	A: 3 patients underwent revision partial repair	Partial repair of massive rotator cuff tears yields comparable short-term results to complete repair.
	B: UCLA 11.2	B: UCLA 29.6	B: 1 patient sustained traumatic retear and underwent revision complete repair	
Kim et al. [18]	Constant 43.6 UCLA 10.5	Constant 74.1 UCLA 25.9	Not reported	Arthroscopic partial repair and margin convergence showed satisfactory short-term outcomes in massive rotator cuff tears
Mori et al. [19]	A: Constant 37.4 ASES 40.8 UCLA 14.3 VAS 7.0 ROM FF 114° ER 27.9° IR 17°	A: Constant 81.1 ASES 94.1 UCLA 32.6 VAS 0.3 ROM FF 160.8° ER 46° IR 11.6°	No complications	In the arthroscopic treatment of irreparable massive rotator cuff tears with low-grade fatty infiltration of infraspinatus, the patch graft showed a lower retear rate (8.3%) than partial repair (41.7%).
	B: Constant 36.3 ASES 41.8 UCLA 13.7 VAS 7.0 ROM FF 110.6° ER 28.1° IR 17°	B: Constant 69.9 ASES 85.7 UCLA 29.8 VAS 1.2 ROM FF 162.3° ER 44.6° IR 11.6°		
Paribelli et al. [20]	Α: UCLA 7.3 VAS 6.9 RC-QOL n.a. ROM FF 83.5° ER 14.5°	Α: UCLA 30.3 VAS 1.3 RC-QOL 81.8 ROM FF 131° ER 41.2°	A: A rupture of the latissimus dorsi tendon was recorded 13 months postoperatively. A reverse total shoulder arthroplasty was performed.	In irreparable rotator cuff tears, arthroscopic-assisted latissimus dorsi tendon transfer and arthroscopic rotator cuff partial repair are both effective ways to treat patients’ symptoms. In younger patients, the first option offers better clinical results.
	B: UCLA 7.6 VAS 6.6 RC-QOL n.a. ROM FF 86.3° ER 15.8°	B: UCLA 20.1 VAS 1.5 RC-QOL 69.3 ROM FF 110° ER 38.4°	B: Not reported	
Shon et al. [21]	ASES 41.97 SST 3.61 VAS 5.13 ROM FF 132.9° ER 35.5° IR 10.6°	ASES 73.78 SST 6.07 VAS 3.16 ROM FF not reported ER not reported IR not reported	Not reported	Arthroscopic partial repair of irreparable massive rotator cuff tears may produce short-term improvement. Fatty infiltration of the teres minor was the identified factor that affected patient-rated satisfaction.

### Clinical and functional outcome variables

Range of motion (ROM) was measured in eight studies (72.7%) [11–13, 15, 16, 19–21], muscle strength in six studies (54.5%) [11, 14, 16, 19–21], and patients’ satisfaction in two studies (18.2%) [15, 21]. The Constant score was used in six (54.5%) of the studies which were included in this review [11, 14–16, 18, 19], whereas the visual analogue scale (VAS) in five studies (45.5%) [12, 15, 19–21] as well as The University of California at Los Angeles (UCLA) Shoulder Score (45.5%) [13, 17–20]. From the rest of the outcome variables, the most commonly used was the American Shoulder and Elbow Surgeons (ASES) score, with a 36.4% rate amongst studies [12, 16, 19, 21], whereas the Disabilities of the Arm, Shoulder and Hand (DASH) Score, subjective shoulder value (SSV), simple shoulder test (SST), and Rotator Cuff Quality of Life (RC-QOL) score were utilized equally in 18.2% [11, 15], 18.2% [14, 15], 18.2% [12, 21], and 18.2% [13, 20], respectively. Finally, the short Western Ontario Rotator Cuff Index was used in one study (9.1%) [16] (Table 3).

All postoperative mean clinical and functional subjective scores, as well as muscle strength of patients treated with arthroscopic partial repair, were found significantly improved, when compared with the respective mean preoperative values in all 11 studies which were included in this review [11–21]. Ten out of 11 studies (90.9%) documented significant postoperative improvement in the ROM of the arthroscopic partial repair-treated patients [11, 13–21], while one study (9.1%) [12] did not illustrate any significant improvement in comparison with the preoperative ROM. In the two studies which evaluated patients’ satisfaction, Heuberer et al. illustrated high postoperative patients’ satisfaction (86%) with the use of partial repair (no significant difference in comparison with the complete repair group as well as the debridement group), whereas Shon et al. showed that only half of the treated patients declared themselves satisfied [15, 21]. The specific preoperative and postoperative mean values of the clinical and functional outcome variables per study can be found in Table 4.

### Repair integrity

Four studies of this review (36.4%) assessed the postoperative re-tear rate of the partial repair [11, 14, 15, 19]. Three of these studies (28.2% of all studies) used diagnostic ultrasonography for their postoperative imaging evaluation [11, 14, 15], while two studies (18.2%) made use of the MRI [15, 19]. Overall, the rate of structural failure of the partial repair was 48.9% amongst these studies [11, 14, 15, 19].

### Radiographic outcomes

Two studies (18.2%) [12, 21] reported the postoperative radiographic progression of osteoarthritis according to Hamada score [24]. According to Shon et al., all patients included in their study were found without any changes in the Hamada score at their final follow-up X-ray when it was compared with the initial preoperative score [21]. On the contrary, 36% of the patients (10 out of 28 patients) who were followed in the study of Cuff et al. had postoperative osteoarthritic progression of one or more Hamada stages [12] (Table 3).

Kim et al. found a small but statistically significant postoperative decrease in the acromiohumeral distance in comparison with the mean preoperative value (from a preoperative mean value of 6.5 mm to a postoperative mean value of 5.9 mm; *p* < 0.001) [18]. However, according to Kim et al., this difference was not clinically significant [18] (Table 3).

## Discussion

The most important finding of this review was that all those studies which evaluated arthroscopic partial repair for the treatment of irreparable MRCT documented significant improvements in all postoperative subjective clinical and functional scores which were measured. All studies showed significant improvement of postoperative muscle strength and almost all of them reported significant improvement in the ROM at the last follow-up assessment. It seems that the rebalanced remaining anterior and posterior parts of the rotator cuff recover shoulder stability, which subsequently allows better function and decreased pain even after a partial non-anatomic repair [25].

In addition, the complication and reoperation rates in our review were found to be very low. Clinical symptoms like persistent pain due to failure of the repair were rather uncommon and only rarely led to a revision surgery. According to these results, arthroscopic partial repair can be chosen as a safe and effective salvage solution in cases where an arthroscopic complete repair cannot be performed. This means that arthroscopic partial repair does not substitute complete anatomic repair, which remains the treatment of choice in cases where the retracted torn tendons can be repaired back to their anatomic footprint.

On the other hand, the re-tear rate of the repair, as estimated by postoperative MRI or ultrasound, was found to be almost 50%. This very high rate of structural failure of the arthroscopic partial repair raises serious concerns regarding the actual value of this treatment. Notwithstanding, the clinical relevance of this finding remains controversial as many patients continue to improve, not only clinically but also functionally (ROM and strength), even when the integrity of the repair has deteriorated. According to Lubiatowski et al., rotator cuff integrity after arthroscopic repair does not seem to affect clinical scores [26]. A possible explanation might be that the reduction of pain-related muscle activity inhibition via arthroscopic debridement, lavage, and intra-articular synovectomy could lead to increased shoulder muscle strength regardless of the success of the repair [11, 27]. Moreover, a possible complete or partial decompression of a tethered suprascapular nerve during the arthroscopic procedure might also contribute to the symptomatic relief of the patient, independently of the longevity of the repair [28].

Kim et al. illustrated that there was a significant decrease in the acromiohumeral distance after surgery [18]. Nevertheless, the importance of this X-ray finding in relation to a possible development of rotator cuff arthropathy has yet to be determined in future studies. While Kim et al. reported no osteoarthritic changes (Hamada score) in any of their patients [18], Shon et al. showed that more than one third of their patients had deteriorated radiographic outcomes [21]. Long-term studies are considered necessary to show whether arthroscopic partial repair results in progression of glenohumeral osteoarthritis or not.

The total number of patients who were treated with arthroscopic partial repair was rather small to extract definite conclusions. In addition, the follow-up varied widely amongst studies, from 2 to 8 years, and no studies reported long-term follow-up. Considering that this is an operative procedure with only short- to mid-term results documented, new studies investigating the long-term outcome of arthroscopic partial repair are required to confirm the therapeutic value of this technique.

The overall quality of the studies was not high and was rated as moderate according to the mean modified Coleman methodology score. Most studies included were characterized by high risk of various potential bias, especially performance and selection bias. In addition, a relative lack of well-designed prospective trials was noted, since there was only one randomized controlled trial [11] and almost all other studies were of level III or IV. Other drawbacks were that some studies assessed heterogeneous populations with significantly different baseline characteristics amongst groups, including patients with different grades of fatty infiltration, size and types of lesions, and number and type of torn tendons. Finally, even the type of the operative procedure which was described as arthroscopic partial repair was not exactly the same. Some physicians performed a medialized repair that allowed for a tension-free repair [29], whereas some other authors used the typical partial repair with margin convergence as initially described by Burkhart et al. [30].

## Conclusions

Arthroscopic partial repair might be a safe and effective alternative treatment for irreparable contracted MRCT, where a complete repair cannot be performed. The methodological quality of the relevant, available literature is low to moderate; therefore, further studies of higher quality are required to confirm these results.

## Abbreviations

Abd: Abduction
Add: Adduction
CMS: Constant-Murley Score
DASH: Disabilities of the Arm, Shoulder and Hand Score
ER: External rotation
FF: Forward flexion
IR: Internal rotation
MRCT: Massive rotator cuff tears
RC-QOL: Rotator Cuff Quality of Life score
RCT: Rotator cuff tears
ROM: Range of motion
SST: Simple shoulder test
SSV: Subjective shoulder value
TW: Text Word
UCLA: University of California at Los Angeles Shoulder Score
VAS: Visual analogue scale
WORC: Western Ontario Rotator Cuff Index
